# Clinical Assessment of Physical Examination Maneuvers for Superior Labral Anterior to Posterior Lesions

**DOI:** 10.1055/s-0037-1606829

**Published:** 2017-10-05

**Authors:** Lyndsay E. Somerville, Kevin Willits, Andrew M. Johnson, Robert Litchfield, Marie-Eve LeBel, Jaydeep Moro, Dianne Bryant

**Affiliations:** 1 Department of Surgery, Schulich School of Medicine & Dentistry, The University of Western Ontario, London, Ontario, Canada; 2 School of Health Studies, Faculty of Health Sciences, The University of Western Ontario, London, Ontario, Canada; 3 Department of Surgery, Division of Orthopaedic Surgery, McMaster University, Hamilton, Ontario, Canada; 4 Department of Clinical Epidemiology & Biostatistics, Faculty of Health Sciences, McMaster University, Hamilton, Ontario, Canada

**Keywords:** shoulder, SLAP, diagnosis, physical examination

## Abstract

**Purpose** Shoulder pain and disability pose a diagnostic challenge owing to the numerous etiologies and the potential for multiple disorders to exist simultaneously. The evidence to support the use of clinical tests for superior labral anterior to posterior complex (SLAP) is weak or absent. The purpose of this study is to determine the diagnostic validity of physical examination maneuvers for SLAP lesions by performing a methodologically rigorous, clinically applicable study.

**Methods** We recruited consecutive new shoulder patients reporting pain and/or disability. The physician took a history and indicated their certainty about each possible diagnosis (“certain the diagnosis is absent/present,” or “uncertain requires further testing”). The clinician performed the physical tests for diagnoses where uncertainty remained. Magnetic resonance imaging arthrogram and arthroscopic examination were the gold standards. We calculated sensitivity, specificity, and likelihood ratios (LRs) and investigated whether combinations of the top tests provided stronger predictions.

**Results** Ninety-three patients underwent physical examination for SLAP lesions. When using the presence of a SLAP lesion (Types I–V) as disease positive, none of the tests was sensitive (10.3–33.3) although they were moderately specific (61.3–92.6). When disease positive was defined as repaired SLAP lesion (including biceps tenodesis or tenotomy), the sensitivity (10.5–38.7) and specificity (70.6–93.8) of tests improved although not by a substantial amount. None of the tests was found to be clinically useful for predicting repairable SLAP lesions with all LRs close to one. The compression rotation test had the best LR for both definitions of disease (SLAP tear present = 1.8 and SLAP repaired = 1.67). There was no optimal combination of tests for diagnosing repairable SLAP lesions, with at least two tests positive providing the best combination of measurement properties (sensitivity 46.1% and specificity 64.7%).

**Conclusion** Our study demonstrates that the physical examination tests for SLAP lesions are poor diagnostic indicators of disease. Performing a combination of tests will likely help, although the magnitude of the improvement is minimal. These authors caution clinicians placing confidence in the physical examination tests for SLAP lesions rather we suggest that clinicians rely on diagnostic imaging to confirm this diagnosis.

Snyder et al 1 first coined the term superior labral anterior to posterior (SLAP) lesion to describe injuries to the superior labral complex that extend from anterior to posterior. They defined these lesions into four types of lesions Types I (degeneration and fraying) through IV (bucket handle tear extending into the biceps tendon), based on arthroscopic findings. Several investigators have since expanded this definition to include Types V to X lesions, 2 3 although these are rarely described in the literature. Of these, Types I and II lesions are most commonly observed in patients. 1 4 5 6

The prevalence of SLAP lesions has been reported to be as high as 26% and as low as 6%. 7 8 9 10 Studies have demonstrated an upward trend in the number of cases of SLAP reported annually. 11 12 Zhang et al 11 demonstrated a 105% increase in the incidence of SLAP repair over a 6-year period (2004–2009). SLAP lesions are rarely found in isolation, most commonly observed in combination with other pathology. 7 10 Snyder et al 7 found that 72% of their patients with SLAP lesions had other associated lesions. Erickson et al 10 showed 38% of SLAP repairs had concomitant procedures. For this reason, SLAP lesions are often difficult to diagnose. Some studies suggest that conservative management of SLAP lesions is unsuccessful in most patients, 13 14 and therefore, it is important that SLAP lesions do not go undiagnosed. In addition, it is important to be able to differentiate SLAP lesions from associated pathology and normal variations of the SLAP complex anatomy. Several normal variants of the superior and anterosuperior labra have been described and have a reported prevalence of 1.5 to 12% and up to 95% in people in their seventh or eighth decade demonstrating a degenerative process. 15 It is important to distinguish between these variants and pathology through examination to create an appropriate surgical plan for the patient. More specifically, SLAP lesion repair requires experience in the surgical technique and potentially special equipment or implants, which can significantly increase surgical time if the repair is unplanned (i.e., the lesion is only found intraoperatively). In addition, repairing a SLAP lesion requires the expertise to do so, which means that a surgeon without such competence may be faced with the decision to either leave the lesion unrepaired (which has the potential to produce symptoms in the future), or to refer the patients for additional surgery with another surgeon. Thus, patient history, physical examination, and diagnostic imaging are important to improve patient management and outcome.

As imaging such as magnetic resonance imaging arthrogram (MRIa) can be invasive and costly, accurate physical examination tests would be an ideal component in the diagnosis of SLAP lesions. Three systematic reviews assessing the accuracy of physical examination maneuvers for SLAP lesions 16 17 18 established that there is no strong evidence to support the use of physical examination tests for SLAP lesions. Most studies did not meet the criteria for internal validity, and therefore report values of sensitivity and specificity that may be biased; most probably overestimating the true validity of these tests. 19 20

Three criteria must be met for a study to be considered robust: (1) the sample of patients must be representative of patients for whom clinicians would face diagnostic uncertainty; (2) the results of the diagnostic test cannot influence which patient undergoes the gold standard test; and (3) the person interpreting the gold standard must be blind to the results of physical exam tests, and other forms of testing. Although not a criteria for internal validity, Mirkovic et al 18 also noted that the majority of clinical tests for SLAP lesions that reported high levels of accuracy were published by the authors who designed the test. Mirkovic et al suggested that these study results should be replicated before endorsing the test for clinical use. All three reviews reporting on these examinations concluded that a methodologically robust study was necessary to inform clinical practice. 16 17 18

The purpose of this study is to determine whether existing physical examination tests can diagnose SLAP lesions accurately in patients who present with shoulder pathology. In addition, we will determine the ability of these tests to distinguish between SLAP lesions that are repairable and those that are not. The findings of this study will inform clinicians which of these physical examination maneuvers are most appropriate to discriminate shoulder pathologies as well as which are most efficient at predicting surgical repair.

## Methods

### Patient Population

Between May 2007 and November 2008, we recruited consecutive patients from four clinicians with a subspecialty in shoulder surgery at two tertiary care orthopaedic centers in Ontario, Canada. All participants presented to clinic for their first consultation to address their complaints of shoulder pain or disability. We excluded patients who were referred for shoulder replacement surgery. All patients gave informed consent. The study was approved by each center's Health Sciences Research Ethics Board.

### Identification of Physical Examination Tests

We identified existing physical examination tests through a systematic review of the literature. In several instances, there were variations in the description of how each test was to be conducted and/or how a positive or negative test result was defined. To reach consensus, we used a modified Delphi process 21 whereby participating surgeons were asked to indicate their preference to include or exclude each test. The survey included the original description of the test and scoring and any modifications. Next, we tallied the results of this survey and included tests for which the majority of surgeons indicated that the test should be included, excluded tests for which the majority of surgeons indicated that the test should be excluded, and produced a second survey for tests for which no majority was reached. A majority was defined as at least four of the five participating surgeons indicating include or exclude.

The second survey presented the results of the first survey and identified tests for which there were discrepancies between surgeons. This survey asked each surgeon to present arguments for why the test should or should not be included in the study and to reaffirm their decision. If, following this second survey, any test was still without a majority decision, we presented surgeons with a document reproducing the argument for and against, and a meeting with the surgeons was held and discussion continued until consensus was reached. In addition, we included tests that were newly reported in the literature at the time of our study. We included the following physical examination tests for SLAP pathology: Speed's test; the anterior slide test; the active compression test; the compression rotation test; biceps load I test; biceps load II test; and the resisted supination external rotation test (see **Appendix A** ).

### Clinical Examination Testing

**Fig. 1:**
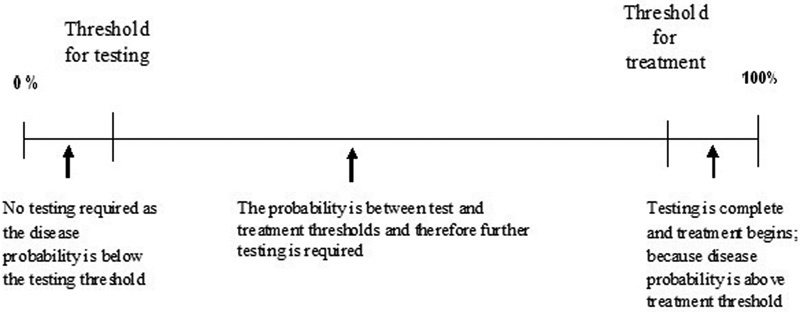
Thresholds in the diagnostic process.

To adhere to the diagnostic process, patients completed a detailed questionnaire prior to their consultation that elicited demographic information, symptoms, and self-reported history of their disease. The physician was not provided with the completed questionnaire to avoid influencing their usual approach to history taking. Instead, the physician completed their usual history including, mechanism of injury, duration of symptoms, history of shoulder injuries, and patient characteristics such as age, occupation, and daily activities. The physician then indicated the pretest probability for each of the eight shoulder pathologies using a diagnostic threshold scale to denote their diagnostic uncertainty (see Fig. 1 ). The eight diagnoses of interest were rotator cuff pathology, acromioclavicular joint pathology, SLAP lesions, other labral lesions, and instability (anterior, posterior, inferior, or multidirectional each represented by a separate scale). Using the scale in Fig. 1 , if the surgeon indicated their uncertainty was below the testing threshold (i.e., certain that the pathology was not playing a role in the patient's complaints) or above the treatment threshold (i.e., certain that the pathology was a contributing factor that no further testing was necessary), they did not perform the tests for that disease. Patients for whom the physician faced uncertainty in the diagnosis (i.e., clinician rated as above the testing but below the treatment threshold) remained as part of the study group for that diagnosis and the physical examination maneuvers specific to that diagnosis were then performed. For example, if the clinician was certain that the patient did not have instability but was uncertain whether the diagnosis was labral pathology, the patient was included in the study group for labral pathology but not for instability. The exclusion of patients from analyses of specific diagnoses if the clinician was certain the patient did not have that particular diagnosis is the key methodological feature that ensures that the results of this study are truly applicable in a clinical setting. To standardize the technique and scoring for each test, we constructed a glossary that was provided to clinicians. Each clinician was required to review the glossary and ensure their method of application matched the description provided. To assist with standardization, we included pictures that illustrate the technique. Further, we used a standardized data collection form that includes the description of how each test is performed and scored. Finally, a graduate student was trained how to perform all physical examination tests and was familiarized with alternative techniques so that they could provide correction if the clinician was performing the test in a manner other than as described in the protocol. A research assistant was present to ensure that all tests are completed and to record the results of the test on the data collection form. We ensured that the physician performing the physical examination tests did not review any available imaging studies or reports before evaluating the patient. This article will discuss the results of the physical examination for the SLAP complex.

### Reference Standard

Arthroscopic examination and MRIa were the main reference standards. The surgeon performed a systematic diagnostic arthroscopy taking care to visualize and evaluate the integrity of all pertinent anatomy and was required to complete a standardized checklist documenting any findings for each structure. We developed this to minimize differences between surgeons in diagnoses due to variations in methods of examination, and to minimize any detection bias should the clinician recall the physical examination or imaging at the time of interpreting the surgical examination.

Although most patients at tertiary centers have surgery, some are not referred for surgery or opt out of recommended surgery. These patients underwent a standardized MRIa as the reference standard. All MRIs had an intra-articular injection of gadolinium done under fluoroscopy. The strength of the MRI magnet was 1.5 T, and the MRI sequences were all protocoled to provide optimal imaging of the pathologies being investigated (axial T1, T1 fat sat, coronal T1 and T1 fat sat, proton density fat sat, T2 fat sat, and sagittal proton density fat sat sequences). All MRIs were done at a university center with substantial expertise in musculoskeletal imaging. Since the literature has shown that MRI alone is not as accurate for diagnosing SLAP tears with reported sensitivities for simple MRI ranging from 43 to 75% 22 23 24 25 26 and specificities between 58 and 70%, 23 24 26 we included arthrogram. There is good evidence to suggest that MRIa is a comparable reference standard to arthroscopy for labral injuries. 27 28 MRIa has been shown to be highly sensitive (100 and 82%) and specific (88 and 100%) for detecting SLAP injuries. 27 28

### Statistical Analysis

To determine sample size, we assumed a sensitivity and specificity of at least 0.85 with a 95% confidence interval (CI) with a width of ± 0.10 29 yielding an estimate of 50 patients tested in each disease category. We inflated the sample size by 10% to account for attrition.

Sensitivity and specificity were calculated for each test individually including 95% CIs around these estimates. These values were used to calculate positive and negative likelihood ratios (LRs). A LR is the likelihood that a test result (positive or negative) is elicited in a patient with the target disorder compared with the likelihood the same test result is elicited in a patient without the target disorder. LRs indicate the extent that a given diagnostic test result will change the odds of having the target disorder. A LR of 1 has little practical significance, as the clinician's impression of the probability of the presence of the target disorder would not change based on this test result. LRs greater than 1 imply that the test result is associated with the disease: the greater the value, the more likely the disorder is present. Conversely, LRs less than 1 indicate that the test result is associated with absence of disease: the closer it is to 0, the less likely the disorder is present.

As we are interested in identifying the tests that accurately diagnose patients who undergo surgery versus those who do not, we repeated this analysis after categorizing patients according to whether existing SLAP pathology was surgically repaired or not repaired. “Repaired” was defined as any manipulation of the SLAP complex that required suturing. Debridement was not considered a repair. For those patients who underwent an MRIa, an experienced surgeon viewed their images and decided whether any existing SLAP pathology was repairable as defined earlier. This was required for 8 of 37 patients who underwent MRIa in this study.

We dummy coded the set of tests to indicate whether one test, two tests, three tests, and so on were positive. We tested whether combinations of tests improve the ability to diagnose. We calculated the sensitivity, specificity, and LR if all tests positive, at least one test is positive and so on. This analysis will determine the appropriate number and combinations of tests for that will provide the greatest clinical yield.

## Results

One hundred and eighty-nine patients participated in this study. Of these 189 patients, 15 patients refused to undergo one of the reference standard tests or canceled their scheduled test; therefore, the remaining 174 patients composed the study sample. Eighty-one patients were not suspected of having SLAP disease (pretest probability of SLAP pathology rated below the testing threshold) and therefore did not undergo SLAP testing. Ninety-three patients underwent physical examination tests for SLAP lesions. Fifty-six (60%) patients underwent surgery as the gold standard, and the remaining had MRIa. There were 64 males and 29 females with an average age of 33.8 ± 14.3. The types of lesions related to the SLAP complex were Types I (13), II (12), III (1), and V (4) lesions. Similar to the reported literature, a majority of these lesions were in combination with other pathology ( Table 1 ). Other pathologies were anterior or posterior labrum lesions, rotator cuff pathology, biceps pathology, and acromioclavicular joint abnormalities.

**Table 1:** Distribution of associated disease in SLAP lesions

SLAP lesion	Biceps tendon pathology	Anterior labrum Bankart	Posterior labrum reverse Bankart	Supraspinatus pathology	Subscapularis pathology	AC OA moderate–severe	Other mild pathology	No other pathology
Type I ( *n* = 13 [14.0%])	3	5	2	5	2	1	5	1
Type II ( *n* = 12 [12.9%])	0	1	1	3	0	1	4	5
Type III ( *n* = 1 [1.1%])	0	0	0	0	0	0	0	1
Type V ( *n* = 4 [4.3%])	0	4	0	0	0	0	1	0
No pathology ( *n* = 63 [67.7%])	4	22	6	9	3	3	25	11

Abbreviations: AC, acromioclavicular; OA, osteoarthritis; SLAP, superior labral anterior to posterior.

The diagnostic validity measures for all of the studied physical examination tests are presented in Table 2 . When using the presence of a SLAP lesion (Types I–V) as disease positive, none of the tests under evaluation was sensitive although they were moderately specific. The active compression test had the largest proportion of patients with a positive test result ( *n* = 34), of these 10 had pathology of the SLAP complex (Types I–V). The compression rotation test only had eight patients with positive results of which half had pathology. When disease positive was defined as the SLAP lesion being repaired, the sensitivity of all tests except for the compression rotation and resisted supination external rotation improved although not by a substantial amount ( Table 3 ). This finding was similar for the specificity of the tests except for the anterior slide and active compression tests. Although this is the case, none of the tests was found to be clinically useful for predicting repairable SLAP lesions as all of the LRs were close to one. There was no optimal combination of tests that improved the sensitivity and specificity for diagnosing repairable SLAP lesions ( Table 4 ).

**Table 2:** Diagnostic values for the physical examination tests for superior labral anterior to posterior complex

Test	Sensitivity	95% CI	Specificity	95% CI	Positive LR	Negative LR
Speed's	27.6	14.7–45.7	71.0	58.7–80.8	0.95	1.02
Anterior slide	20.0	9.5–37.3	73.8	61.6–83.2	0.76	1.08
Active compression	33.3	19.2–51.2	61.3	48.9–72.4	0.86	1.09
Compression rotation	13.8	5.5–30.6	92.6	82.5–97.1	1.8	0.93
Biceps load I	10.3	3.6–26.4	87.0	75.6–93.6	0.80	1.03
Biceps load II	27.6	14.7–45.7	77.8	65.1–86.8	1.24	0.93
Resisted supination ER	14.3	5.7–31.5	80.8	68.1–89.2	0.743	1.06

Abbreviations: CI, confidence interval; ER, external rotation; LR, likelihood ratio.

**Table 3:** Diagnostic values for the physical examination tests for superior labral anterior to posterior complex repaired versus not repaired

Test	Sensitivity	95% CI	Specificity	95% CI	Positive LR	Negative LR
Speed's	29.3	20.2–40.4	70.6	46.9–86.7	1.00	1.00
Anterior slide	25.7	17.1–36.7	82.4	59.0–93.8	1.46	0.90
Active compression	38.7	28.5–50.0	70.6	46.9–86.7	1.32	0.87
Compression rotation	10.5	5.2–20.0	93.8	71.7–98.9	1.67	0.96
Biceps load I	11.9	6.2–21.8	87.5	64.0–96.5	0.96	1.01
Biceps load II	25.4	16.5–36.9	81.3	57.0–93.4	1.35	0.92
Resisted supination ER	16.9	9.7–27.8	81.3	57.0–93.4	0.90	1.02

Abbreviations: CI, confidence interval; ER, external rotation; LR, likelihood ratio.

**Table 4:** Diagnostic validity of the combination of physical examination maneuvers

	Sensitivity	95% CI	Specificity	95% CI	Positive LR	Negative LR
At least one positive	58.7	47.4–69.1	47.1	26.2–69.0	1.11	0.88
At least two positive	46.1	35.3–57.2	64.7	41.3–82.7	1.31	0.83
At least three positive	23.7	15.5–34.4	76.5	52.7–90.4	1.01	1.00
At least four positive	13.2	7.3–22.6	88.2	65.7–96.7	1.12	0.98
At least five positive	5.3	2.1–12.8	94.1	73.0–99.0	0.90	1.01
At least six positive	4.0	1.4–11.0	100.0	81.6–100.0		0.96
All positive	1.3	0.3–7.1	100.0	81.6–100.0		0.99

Abbreviations: CI, confidence interval; LR, likelihood ratio.

## Discussion

Our study demonstrates that the physical examination tests for SLAP lesions are poor diagnostic indicators of disease. No test had a value of sensitivity exceeding 40%. When using SLAP repaired as disease positive, the active compression test had the greatest combination of test properties with a sensitivity of 38.7% (95% CI: 28.5–50.0) and a specificity of 70.6% (95% CI: 46.9–86.7). The compression rotation test was found to be the most clinically useful with a LR approaching two.

Our values of sensitivity and specificity are lower than most reported values in the existing literature. There are several reasons that may explain the inconsistency between our study and others. One reason is that our study includes patients for whom there is diagnostic uncertainty. This is an important feature of the present study methodology—patients with the full spectrum of the disease of interest should be included in the sampling frame, including those with and without concomitant pathology, and those with other shoulder pathology that present with similar symptoms. In the existing literature, a substantial proportion of studies include patients for which the clinician does not face diagnostic uncertainty. For example, in a study by Kibler 30 to validate the anterior slide test for diagnosing SLAP lesions, the reported value of specificity was extremely encouraging (91.5%), but the study included a large proportion (44%) of patients who did not have shoulder complaints or were considered to have normal shoulders. Including patients known to be disease free will overestimate specificity by increasing the proportion of patients with a negative test. In contrast, we found the specificity of the anterior slide test to be 73.8%.

Another common error that we identified in many of the published studies is the exclusion of patients who do not undergo surgery. By limiting the sample to only those who undergo surgery, a study excludes those patients for whom surgery was not recommended or those who elect not to undergo surgery (all of whom are likely to have less severe pathology). By excluding these patients, one increases the proportion of patients who are more likely to have a positive test (since they have more severe pathology) thereby overestimating the sensitivity. For example, Bennett 31 reported the Speed's test to be highly sensitive for diagnosing SLAP lesions (90.0%). Ardic et al 32 who used simple MRI as the gold standard reported a less promising sensitivity of 60.0%. In contrast, we included patients undergoing either surgery or MRIa and found the sensitivity of this test to be 27.6%.

Although not a criteria for internal validity, it has been noted that there is a tendency for articles reporting on the development of a physical examination test to report high levels of accuracy. 33 This phenomenon has been demonstrated in studies reporting on the validity of physical examination tests for SLAP lesions. 18 We selected three recently developed tests—the biceps load I, 6 biceps load II, 5 and the resisted supination external rotation tests 34 —in an attempt to replicate the encouraging results found by their originators. Reports by the test developers have demonstrated very high values of sensitivity (90.9, 89.7, and 82.8%, respectively). Our results were not nearly as positive with values of sensitivity of 10.3, 27.6, and 14.3%, respectively. These results improved when we defined disease positive as surgically repaired SLAP pathology, but they still did not exceed 30%. This demonstrates the need to replicate the results of studies reporting high values of sensitivity and specificity as they may lead a clinician to inappropriately adopt these tests into practice. Until these tests are critically evaluated, investigators should refrain from developing new tests. In addition, clinicians should be aware of the limitations of the literature reporting on the validity of these physical examinations in arriving at timely and appropriate diagnoses, as well as successful subsequent management of these lesions.

Surgical management is the most common therapy for treatment of SLAP lesions. Arthroscopic debridement yields inconsistent result; therefore, SLAP lesion repair has become standard treatment. Various methods of fixation have led to successful functional results; however, some patients, in particular active patients, report lower level of satisfaction in this procedure. 35 Recently, shoulder surgeons have been performing biceps tenodesis as a treatment option for these patients. 35 In addition, this procedure can be used as a salvage procedure for failed SLAP repairs. Diagnostic tools need to be able to identify these lesions to avoid a delay in treatment. In addition, it is necessary that any examination should be sensitive enough to differentiate between concomitant pathologies considering the majority of SLAP lesions are seen with associated lesions. For instance, if a patient is diagnosed with a rotator cuff tear, and gets booked for a rotator cuff repair, a particular amount of time is set aside for this intervention. If, during the procedure, a SLAP lesion is unexpectedly encountered, this can add significant surgical time to the procedure. Alternatively, if the patient is booked for a rotator cuff tear with a possible SLAP repair, and they do not have the SLAP lesion, the surgeon will have committed surgical time that could have been used elsewhere. Therefore, an undiagnosed SLAP lesion can result in either running behind or ahead of schedule in the operating room resulting in an inefficient use of resources. In some cases, depending on the institution or even the health care system, penalties are applied to the clinician's practice for running over the scheduled operating room time.

This study involved four surgeons in two different cities in Ontario, Canada, which will increase the applicability of the results. Since this project is an initiative of surgeons who are members of a large national group, there is enormous potential for knowledge transfer, in that surgeons will use the results to guide practice, teach medical students, residents, and fellows according to their practice. With the standardization of tests, a more research-friendly atmosphere can be created.

## Limitations

The limitations of this study include the potential for detection bias since the surgeon who completes the physical examination will also complete the surgical evaluation. We have minimized the potential for this source of bias by creating a standardized protocol for diagnostic shoulder arthroscopy that all surgeons will perform, so that all structures are investigated carefully and reported in a standardized fashion. In addition, the time delay (average 4 months) between the clinical examination tests and surgical evaluation and the large volume of patients being included in this study reduces the probability that the surgeon will remember the results of the physical examination at the time of surgical evaluation. Finally, although we recruited the estimated sample size requirement, the CIs for test measurement properties are wide, and therefore, a larger sample size may provide more precise estimates of the accuracy of these tests.

## Conclusion

Based on these study results, clinicians must understand that no test in isolation is sufficient to diagnose a patient with a SLAP lesion. Performing a combination of tests will more likely help a clinician diagnose SLAP lesions, although the magnitude of the improvement is minimal. Based on the study results, these authors would caution clinicians who place confidence in the physical examination tests for SLAP lesions. In addition, clinicians should be aware of the pitfalls of the majority of published studies that evaluate the diagnostic validity of shoulder examination tests for SLAP lesions. Clinicians must ensure that tests have undergone rigorous testing before adopting them into practice. We suggest that clinicians rely on diagnostic imaging to confirm this diagnosis as none of the physical examination maneuvers was found to be clinically useful.

## Appendix A Physical examination test list

**Speed's Test**

The patient's arm is placed with the forearm in full supination and at 90 degrees of shoulder elevation. The examiner then applies a downward force to the arm and the patient is asked to resist the force.

*Positive* : The test is positive if the patient experiences pain in the anterior proximal portion of the shoulder during the application of the force.

**Compression Rotation Test**

The patient is in the supine position with the shoulder abducted to 90 degrees and the elbow flexed at 90 degrees. A compression force is applied to the humerus, which is then rotated.

*Positive* : The test is positive if labral tissue is felt to catch and snap during the test.

**Anterior Slide Test**

The patient is examined either standing or sitting with their hands on their hips with thumbs pointing posteriorly. One of the examiner's hand is placed across the top of the shoulder from the posterior direction with the last segment of the index finger extending over the anterior aspect of the acromion at the glenohumeral joint. The examiner's other hand is placed behind the elbow and a forward and slightly superiorly directed force is applied to the elbow and upper arm. The patient is asked to push back against the force.

*Positive* : The test is positive if the patient has pain localized to the front of the shoulder under the examiner's hand and/or a pop or click in the same area or if the maneuver reproduces symptoms that occur during their overhead activities.

**Active Compression Test/O'Brien's Test**

The examiner stands behind the standing patient and the patient forward flexes the affected arm 90 degrees with the elbow in full extension. The patient then horizontally adducts the arm 10 to 15 degrees medial to the sagittal plane of the body. The arm was then internally rotated so that the thumb is pointing downward. The patient resists as the examiner applies a uniform downward force to the arm. With the arm in the same position, the palm is then fully supinated and the maneuver is repeated.

*Positive* : The test is positive if pain is elicited with the first maneuver and is reduced or eliminated with the second maneuver.

**Biceps Load Test I**

The patient lies in the supine position. The examiner sits at a right angle and at the same height to the patient on the side of the affected shoulder. The examiner gently grasps the patient's wrist and elbow. The arm is abducted at 90 degrees with the forearm in the supinated position with the elbow flexed to 90 degrees. The patient relaxes and an apprehension test is performed (taking arm into full external rotation). When the patient becomes apprehensive during the external rotation of the shoulder, the external rotation is stopped. The patient is then asked to flex the elbow, while the examiner resists (on the same plane as the patients arm so as not to change the degree of abduction and external rotation) the flexion with one hand and asks how the apprehension has changed if at all. The test is repeated and the patient is instructed not to pull the whole upper extremity, just bend the elbow against the resistance.

*Positive* : The test is positive if the apprehension has not changed or if the shoulder becomes more painful.

**Biceps Load Test II**

The patient lies supine. The examiner sits adjacent to the patient on the same side as the affected arm grasping the wrist and elbow gently. The arm is elevated to 120 degrees and externally rotated to its maximal point with the elbow in 90 degrees of flexion and the forearm is supinated. The patient is asked to flex the elbow against the examiner's resistance.

*Positive* : The test is positive if the patient complains of pain during the resisted elbow flexion or if the patient complains of more pain from the resisted elbow flexion regardless of the degree of pain before the elbow flexion maneuver. The test is negative if pain is not elicited by elbow flexion or if the preexisting pain during the elevation and external rotation of the arm is unchanged or diminished by the resisted elbow flexion.

**Resisted Supination External Rotation Test**

The patient is in the supine position with the scapula near the edge of the table. The examiner stands at the patient's side, supporting the affected arm at the elbow and hand. The arm starts in 90 degrees abduction with the elbow flexed 65 to 70 degrees and the forearm in neutral or slight pronation. The patient is asked to attempt to supinate the hand with maximal effort as the examiner resists and gently externally rotates the shoulder joint to maximal external rotation. The patient is asked to describe the symptoms at maximal external rotation.

*Positive* : The test is positive if the patient describes anterior or deep shoulder pain, clicking or catching in the shoulder, or reproduction of symptoms that occur during a throwing motion. The test is negative if the patient describes posterior pain, apprehension, or no pain.
